# The oral selective oestrogen receptor degrader (SERD) AZD9496 is comparable to fulvestrant in antagonising ER and circumventing endocrine resistance

**DOI:** 10.1038/s41416-018-0354-9

**Published:** 2018-12-17

**Authors:** Agostina Nardone, Hazel Weir, Oona Delpuech, Henry Brown, Carmine De Angelis, Maria Letizia Cataldo, Xiaoyong Fu, Martin J. Shea, Tamika Mitchell, Jamunarani Veeraraghavan, Chandandeep Nagi, Mark Pilling, Mothaffar F. Rimawi, Meghana Trivedi, Susan G. Hilsenbeck, Gary C. Chamness, Rinath Jeselsohn, C. Kent Osborne, Rachel Schiff

**Affiliations:** 1 Lester & Sue Smith Breast Center, Baylor College of Medicine, Houston, TX 77030 USA; 2 Department of Medical Oncology, Dana Farber Cancer Institute, Boston, MA 02210 USA; 3 Bioscience, Oncology, IMED Biotech Unit, AstraZeneca, Cambridge, UK; 4 Dan L. Duncan Comprehensive Cancer Center, Baylor College of Medicine, Houston, TX 77030 USA; 5 Quantitative Biology, Discovery Science, IMED Biotech Unit, AstraZeneca, Cambridge, UK; 6 Department of Medicine, Baylor College of Medicine, Houston, TX 77030 USA; 7 Department of Pharmacy Practice and Translational Research, University of Houston College of Pharmacy, Houston, TX USA

**Keywords:** Breast cancer, Cancer

## Abstract

**Background:**

The oestrogen receptor (ER) is an important therapeutic target in ER-positive (ER+) breast cancer. The selective ER degrader (SERD), fulvestrant, is effective in patients with metastatic breast cancer, but its intramuscular route of administration and low bioavailability are major clinical limitations.

**Methods:**

Here, we studied the pharmacology of a new oral SERD, AZD9496, in a panel of in vitro and in vivo endocrine-sensitive and -resistant breast cancer models.

**Results:**

In endocrine-sensitive models, AZD9496 inhibited cell growth and blocked ER activity in the presence or absence of oestrogen. In vivo, in the presence of oestrogen, short-term AZD9496 treatment, like fulvestrant, resulted in tumour growth inhibition and reduced expression of ER-dependent genes. AZD9496 inhibited cell growth in oestrogen deprivation-resistant and tamoxifen-resistant cell lines and xenograft models that retain ER expression. AZD9496 effectively reduced ER levels and ER-induced transcription. Expression analysis of short-term treated tumours showed that AZD9496 potently inhibited classic oestrogen-induced gene transcription, while simultaneously increasing expression of genes negatively regulated by ER, including genes potentially involved in escape pathways of endocrine resistance.

**Conclusions:**

These data suggest that AZD9496 is a potent anti-oestrogen that antagonises and degrades ER with anti-tumour activity in both endocrine-sensitive and endocrine-resistant models.

Oestrogen receptor alpha (ER) and its ligand oestrogen (E2) are important drivers of breast cancer initiation and progression. Over two-thirds of breast cancers express the ER transcription factor, and in most ER-positive (ER+) tumours, ER remains a key driver and a therapeutic target even after development of resistance to initial endocrine therapy^1^. Endocrine therapies, aiming to reduce ER activity, encompass selective ER modulators (SERMs) such as tamoxifen which bind to ER and modulate its functions; strategies that systemically reduce E2 level in order to deprive the receptor of its ligand using aromatase inhibitors (AIs) or ovarian ablation; and selective ER degraders (SERDs) such as fulvestrant which function as more complete antagonists and degrade ER protein. Although endocrine therapy is highly effective, intrinsic and acquired resistance are still common in both early and advanced settings, and in the metastatic stage almost all patients who initially respond to the therapy eventually progress and succumb to the disease^2^.

Fulvestrant is the first and only SERD that has been clinically approved for the treatment of postmenopausal patients with ER+ metastatic breast cancer after progression on tamoxifen or AIs. A number of studies have shown that fulvestrant treatment in patients was unable to achieve complete ER degradation^3,4^. Although it is possible that doses higher than 500 mg of fulvestrant may achieve better ER degradation, its pharmacodynamics and intramuscular route of administration limit the amount of fulvestrant that can be given to patients^5,6^. Therefore, there is a compelling clinical need for oral SERDs with higher bioavailability, increased receptor degradation capability, enhanced antagonist activity, and potential use in premenopausal patients who have high oestrogen levels.

AZD9496 is an oral SERD, and it has been selected by direct screening of drug-like ER ligands^7^. AZD9496 has been shown to potently antagonise and degrade ER in preclinical studies with MCF7 ER+ breast cancer cell line and xenograft models as well as in patient-derived xenografts harbouring an *ESR1* mutation^7–9^. Moreover, combining an inhibitor of the phosphoinositide 3-kinase (PI3K) pathway or of cyclin-dependent kinase-4/6 (CDK4/6) with AZD9496 led to an enhanced tumour inhibitory effect^8^. More recently, a phase I clinical trial of AZD9496 was reported, and AZD9496 was well tolerated and had an acceptable safety profile^10^. In addition, a number of heavily pre-treated patients experienced prolonged disease stabilisation^10^. Currently, there is an ongoing open-label, randomised, multicentre window-of-opportunity pharmacodynamics study (NCT03236974) to compare and evaluate the biological effects of AZD9496 versus fulvestrant.

In the present study, we have investigated the activity of AZD9496 across a panel of endocrine-sensitive and -resistant breast cancer cell lines and xenograft models and compared the efficacy of AZD9496 with fulvestrant. We demonstrate that AZD9496 robustly reduces ER levels and inhibits the growth of both endocrine-resistant and -sensitive cell line models in vitro. AZD9496 significantly delays ER-dependent endocrine-resistant tumour growth in vivo. Importantly, when compared to fulvestrant, AZD9496 exhibited overall similar inhibitory activity on ER signalling and on growth of tumour cells and xenografts. Analysis of endocrine-sensitive and -resistant xenograft tumours indicates that AZD9496 antagonises ER regulation of transcription, including E2-induced and -repressed genes involved in cell growth and potentially in escape pathways of endocrine resistance.

## Materials and methods

### Cell lines, establishment of resistant lines, and reagents

MCF7 and T47D ER+ breast cancer cell lines and their corresponding derivatives resistant to oestrogen deprivation or tamoxifen (EDR and TamR, respectively) were grown as previously described^11,12^. To establish fulvestrant resistance (FulR), MCF7 and T47D parental cells were maintained continuously in the presence of fulvestrant (10^–7^ M) for at least 6 months in phenol red-free media in the presence of 10% charcoal-stripped (cs) foetal bovine serum (FBS). Parental cells were cultured in Dulbecco's modified Eagle's medium (600MPE and MDA-MB-415) or RPMI (T47D, MCF7, and ZR75-1) media with 10% FBS and 1% penicillin/streptomycin and glutamine. All cell lines were authenticated at the MD Anderson Characterized Cell Line Core Facility and were tested to be mycoplasma-free by MycoAlert™ Mycoplasma Detection Kit (Lonza, Houston, TX). AZD9496 (AstraZeneca, UK) was dissolved in dimethyl sulphoxide (DMSO). The 17ß oestradiol (E2), 4-hydroxy tamoxifen (for all in vitro studies, from Sigma (St Louis, MO)), and fulvestrant (AstraZeneca) were dissolved in ethanol. Tamoxifen citrate (Sigma) was used for all in vivo treatments as previously described^13^.

### Cell growth assays

Parental and resistant cells were oestrogen-deprived (ED) in phenol red-free medium containing 5% cs-FBS (ED-medium) for 72 h, then plated in 96-well plates in ED-medium for another 24 h before beginning additional treatments. A reference plate was fixed at day 0, and endocrine treatments of E2 (10^–9^ M), ED (continued ED-medium), tamoxifen (10^–7^ M), fulvestrant (10^–7^ M, or as indicated), or AZD9496 (10^–7^ M, or as indicated) were added. Media were replaced after 3 days, and after 6 days plates were fixed and stained with methylene blue (Sigma)^14^. The percentage of growth was determined as previously described^15^ using the formula [(cell number at day 6 – cell number at day 0) Treatment]/[(cell number at day 6 – cell number at day 0) Control (DMSO or as specified in figure legend)]. For all treatment groups, cells were plated in quadruplicate.

### Immunoblotting assays

Cells were plated in original media or in ED-medium following by endocrine treatment for 48 h as indicated. Cells and xenograft tissue were lysed and processed as previously described^16,17^. Immunoblotting with the specific primary antibodies was performed according to the manufacturer’s instructions. Primary antibodies used were: β-actin (Cell Signaling Technology), ERα 6F11 (Abcam, Fremont, CA), and progesterone receptor (PR) (Santa Cruz Biotechnology, Santa Cruz, CA). Western blots were performed at least two independent times. Images were acquired as previously described^11,14^ or by using ChemiDoc Touch Imaging System and Image Lab software (BioRad, Hercules, CA).

### ERE-luciferase reporter assays

Cells after 3 days in ED-medium were transfected overnight with oestrogen responsive element (ERE)–luciferase and β-galactosidase constructs using X-treme GENE HP-DNA transfection reagent (Invitrogen) in phenol red-free Opti-MEM reduced-serum medium (HyClone, Logan, UT) as previously described^18^. Cells were then treated for additional 24 h with ED, E2 (10^–9^ M), or 10% FBS, plus tamoxifen (10^–7^ M), fulvestrant (10^–7^ M), or AZD9496 (10^–7^ M). Relative luciferase activity was determined and analysed as previously described^18^.

### Xenograft studies

All animal care was in accordance with institutional guidelines. All studies were conducted using ovariectomised 5–6-week-old athymic mice (Harlan Sprague Dawley, Madison, WI).

#### MCF7 parental study

MCF7 parental cells were injected into both sides of mice supplemented with an oestrogen pellet as previously described^19^. When one of the two tumours reached 200 mm^3^, mice were randomised to six arms including: (i) continue E2 plus vehicle, (ii) E2 plus fulvestrant (4 × 5 mg/mouse in 10 days), (iii) E2 plus AZD9496 (5 mg/kg daily), (iv) oestrogen deprivation (ED) by removing E2 pellet plus vehicle, (v) ED plus fulvestrant, and (vi) ED plus AZD9496. Tumours were harvested when the two tumours reached 1000 mm^3^ (E2 group) or after 8 days of treatment.

#### MCF7 TamR model study

The MCF7 TamR xenograft tumours were generated and maintained as previously described^20^. Mice were pre-treated with tamoxifen for 48 h (500 µg subcutaneously (s.c.)) and transplanted on both sides with tumours derived from two independent donors. When at least one of the two tumours reached 200 mm^3^ in volume, mice were randomised to continue tamoxifen (Tam) as control or stop tamoxifen and switch to vehicle^8^, fulvestrant (5 mg/mouse once a week s.c., as previously described^21^, with an extra dose in the first week), or AZD9496 (0.5, 5, or 50 mg/kg by oral gavage daily). Tumour volumes were measured weekly as previously described^13^. Short-term treatment (10 days) was conducted for biomarker analysis, and long-term treatment (until tumour reached 1000 mm^3^) was conducted to assess progression-free survival. All tumours were harvested 24 h post fulvestrant, and 4 h post vehicle or AZD9496.

#### MCF7 EDR study

The E2-stimulated MCF7 parental xenograft tumours that were initially sensitive to ED resumed growth after almost 1 year in the absence of E2. These tumours were then transplanted into mice without E2 supplementation and grown for several generations in order to stabilise an MCF7 EDR model. In this study, mice bearing a unilateral 200 mm^3^ MCF7 EDR transplantable tumour were randomised to vehicle, fulvestrant (5 mg/mouse), or AZD9496 (10 mg/kg). All tumours were harvested when tumours in the ED control arm reached 1000 mm^3^ in volume.

### Immunohistochemistry (IHC)

Formalin-fixed, paraffin-embedded tumour sections were subjected to immunohistochemical staining of ER as previously described^17^. Tumours were scored by ER H-score (percentage of positive cells × intensity of the staining) independently by two observers (a pathologist and a researcher).

### RNA isolation, cDNA, and real-time PCR analysis

Total RNA was extracted, and reverse transcribed as previously described^22^. Quantitative real-time PCR amplification conditions and primers for *ESR1*, *PGR*, and *β-actin* have been described previously^22^. The relative fold differences in gene expression were calculated by the ΔΔCt method with *β-actin* as a normalisation control.

### Targeted gene expression of xenograft tumours

Targeted gene expression was performed using a 48 × 48 or 96 × 96 Fluidigm dynamic array (Fluidigm, San Francisco CA, USA) and Taqman primers (Thermo Scientific, Waltham, MA). Following the manufacturer’s instructions, 50 ng of total RNA from xenograft tumours were reverse transcribed using a high-capacity complementary DNA (cDNA) reverse transcription kit (Thermo Scientific) and pre-amplified with a Taqman PreAmp master mix (Thermo Scientific) for 14 cycles with 45 selected ER target gene primers. The Fluidigm Array was then primed and loaded on an IFC Controller and quantitative PCR (qPCR) experiments were run on the Biomark System, using the standard Default_10 min_HotStart protocol or M96_default protocol for 48 × 48 or 96 × 96 chips, respectively. Data were collected and analysed using the Fluidigm Real-Time PCR Analysis software to generate the Ct values. Gene expression calculations were performed in Jmp^®^12.0.1, and data represented in TIBCO^TM^ Spotfire^®^ 6.5.2. The Ct values of target genes were normalised to the average of housekeeping genes. The expression of each individual gene in each treatment group was then normalised to its respective expression in the control group to calculate log2 fold change in gene expression (negddCt): the MCF7 TamR model (Fig. 4d) was compared to the tamoxifen group, while the MCF7 endocrine-sensitive model (Fig. 2d) was compared to the vehicle group in each E2 or ED condition.

### Statistical analysis

**Fig. 1:**
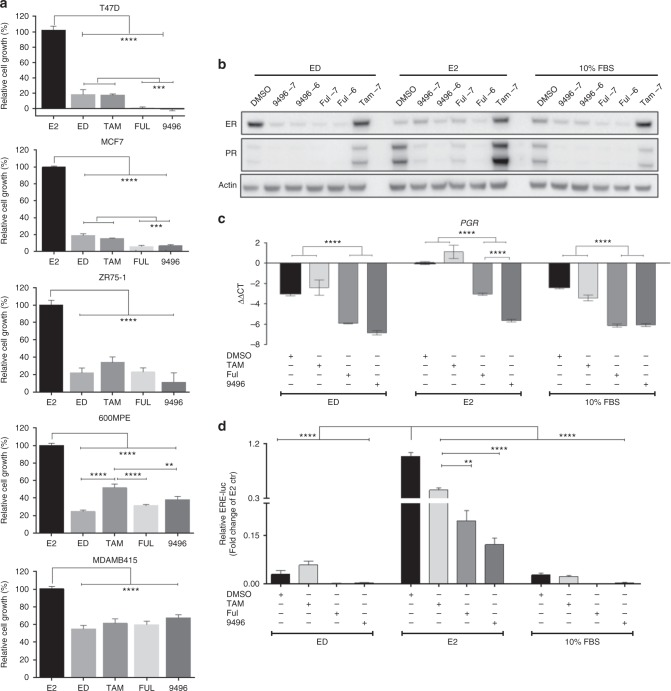
AZD9496 is comparable to fulvestrant in endocrine-sensitive ER +  cells. **a** Cell growth assay of T47D, MCF7, ZR75-1, 600MPE, and MDA-MB-415 parental cells treated for 6 days with different endocrine treatments. **b** Immunoblot for ER expression and signalling (PR) in MCF7 cells in the presence of 5% cs-FBS (ED-medium, ED), ED+E2, or 10% FBS and two different concentrations 10^–6^ M (–6) and 10^–7^ M (–7) of fulvestrant or AZD9496. **c** mRNA levels of progesterone receptor (*PGR*) in MCF7 cells were assessed using real-time quantitative PCR (RT qPCR). The mRNA expression was normalised to the actin housekeeping gene, and expression levels are presented as –ΔΔCT compared with E2 control. **d** ERE-luciferase activity assay in MCF7 cells treated by ED, ED+E2, or 10% FBS for 24 h. SEM are shown (*n* = 3); **p < 0.01; ***p < 0.001; ****p < 0.0001

Cell growth and in vitro RNA expression were analysed within each E2-stimulated or endocrine therapy group using one- or two-way analysis of variance (ANOVA) with the Bonferroni post hoc test using GraphPad Prism version 6.05 (GraphPad, La Jolla, CA). Error bars on plots represent ± standard error of the mean (SEM) (Figs. 1, 3).

**Fig. 2:**
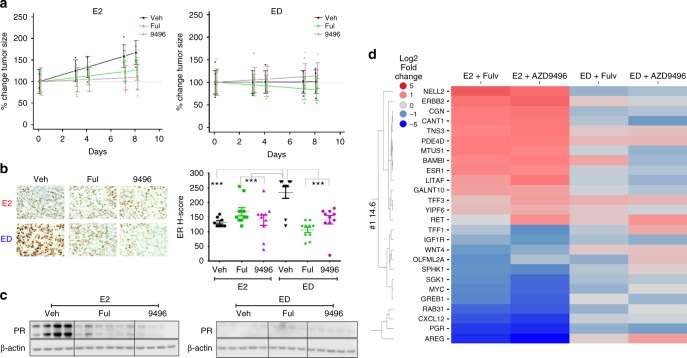
AZD9496, like fulvestrant, inhibits tumour growth and ER signalling in vivo in the presence of E2. Mice bearing MCF7 xenografts in the presence of exogenous E2 were randomised to continued E2 or switched to ED, all plus vehicle (Veh), fulvestrant (Ful), or AZD9496 (9496), and monitored for 8 days. **a** Tumour growth curve of MCF7 cells in E2 or ED condition in the presence of vehicle, fulvestrant, or AZD9496 (*n* = 4–5). Graphs show the model-predicted values and 95% confidence intervals at each time point. For display purposes, predicted values have been ‘normalised’ by dividing predicted values and 95% confidence limits by the day 0 treatment group predicted value. Measurements of individual tumour sizes from each mouse were normalised in the same manner and plotted. **b** Representative ER IHC and quantification of ER protein level by IHC using H-score. **c** Immunoblot of ER downstream target gene PR and β-actin control in available E2 or ED-treated tumours. **d** Log2 fold change of the average gene expression in tumours from each treatment group. Heat map of genes differentially expressed in the E2 treated groups. SEM are shown; *p < 0.05; **p < 0.01; ***p < 0.001

For the short-term (8 days) xenograft growth study of MCF7 parental (endocrine-sensitive cells) experiment (Fig. 2), tumour sizes from the right and left side were summed to generate a single measurement of tumour burden at each time point for each mouse. For each endocrine treatment group (E2, ED), a mixed general linear model was used to model the effect of drug (SERD) treatment (categorical), time (continuous, days), and their interaction as fixed effects on tumour size. A random intercept was used to account for the effect of the starting tumour size for each mouse. Differences in tumour growth were realised as treatment-specific slopes and tested by the interaction term. In the event that the ‘treatment × time’ interaction effect was significant, pairwise comparisons were used to identify the groups that differ. The *p* values for the pairwise comparisons were adjusted by the Holm method to account for multiple comparisons within the endocrine treatment groups. For in vivo long-term TamR xenograft growth studies (Fig. 4), the average size of both tumours in each mouse was used for the statistical analysis. Time to tumour progression (tumour tripling) was summarised with Kaplan–Meier curves and compared by generalised Wilcoxon tests followed by pairwise comparisons with *p* value adjustment to compare the difference between treatments. Analyses and graphs were prepared using R (version 3.3.1 and the survival package). ER protein expression (H-score) after short- (Fig. 2b) or long-term (Fig. 4f) treatments was tested by one- or two-way ANOVA using GraphPad Prism (GraphPad). ER H-score for the short-term TamR experiment, with multiple AZD9496 doses, was tested using the ‘Cuzick nonparametric test for trend’^23^ (Fig. 4b, c).

To generate the heat maps, statistical analysis was performed in JMP software and SAS9.2. A two-sided pairwise *t*-test was performed in JMP for the MCF7 endocrine-sensitive model (Fig. 2) to identify genes significantly modulated upon treatment (Vehicle versus AZD9496 or fulvestrant; and AZD9496 versus fulvestrant or Vehicle) in each E2/ED condition. The gene expression analysis of the MCF7 TamR model (Fig. 4) was executed in SAS9.2. A generalised linear model with a random effect for mouse was used, with the Kenward–Roger correction for degrees of freedom to appropriately deal with a small amount of missing data. Treatment was treated as a categorical variable. In order to take into account left and right tumour replicate samples from animals, a model where left/right as a fixed effect nested within animals was used. Interaction terms in this two-way model were initially explored but found to be non-significant. *PGR* missing values were artificially replaced by values on the limit of detection in order to be able to represent PGR down-regulation after treatment. All main effects were tested, but only treatment comparisons were of interest. Pairwise comparisons between all treatment levels were calculated, therefore Tukey’s HSD (honestly significant difference) adjusted *p* values are reported.

## Results

### AZD9496 is comparable to fulvestrant in inhibiting cell growth and reducing ER levels and activity in endocrine-sensitive cell line models

We first explored the efficacy of AZD9496 in a panel of ER+ parental (endocrine-sensitive) breast cancer cell lines in comparison with other endocrine therapies including fulvestrant. Cell growth changes were assessed for T47D, MCF7, ZR75-1, 600MPE, and MDA-MB-415 cells maintained in ED-medium and treated with E2 (control), ED alone (to mimic an aromatase inhibitor), ED plus tamoxifen, fulvestrant, or AZD9496. Compared to E2 treatment, endocrine therapy (ED, tamoxifen, fulvestrant, and AZD9496) significantly inhibited the growth of all five parental cell lines, although the degree of growth inhibition substantially varied across cell lines with T47D being the most sensitive (80 to 100% inhibition by various endocrine therapies) and MDA-MB-415 the least sensitive (<50% inhibition by all endocrine therapies) (Fig. 1a). Importantly, fulvestrant and AZD9496 exerted similar degrees of growth inhibition in all parental lines tested, and both were more potent compared to ED and tamoxifen in the two most endocrine-sensitive models, T47D and MCF7.

We next used the MCF7 model to compare the efficacy of AZD9496 and fulvestrant in reducing the protein levels of ER as well as its downstream gene product *PR* under ED alone or in the presence of oestrogen (10^–9^ M E2 or 10% FBS) (Fig. 1b). As expected, due to ligand-dependent degradation of the receptor, ER levels were markedly reduced in the presence of E2, compared to ED or tamoxifen. AZD9496 and fulvestrant significantly decreased ER levels, with greater degradation under ED conditions (Fig. 1b). Moreover, both SERDs, but not tamoxifen, effectively inhibited ER transcriptional activity as measured by messenger RNA (mRNA) and protein levels of PR (Fig. 1b, c). A similar decrease in ER transcriptional activity was observed by ERE-luciferase reporter assay, although residual ER activity was observed with both SERDs in the E2 condition (Fig. 1d) and higher concentrations of the two SERDs were needed to inhibit cell growth in the presence of E2, as shown by the increasing half-maximal inhibitory concentrations (IC50) of AZD9496 and fulvestrant in the presence of escalating concentrations of E2 (Supplementary Table 1). These in vitro results suggest that AZD9496 is comparable to fulvestrant in endocrine-sensitive cells.

### AZD9496 inhibits tumour growth and ER signalling in vivo in the presence of E2 in the naive setting

We next tested the effects of AZD9496 on tumour growth and ER levels and activity in vivo in the presence and absence of E2 using the MCF7 xenografts. Mice bearing MCF7 xenograft tumours that were developed in the presence of E2 were randomised to continued E2 or switched to ED in the presence of vehicle, fulvestrant, or AZD9496 (Fig. 2a). In the presence of E2, both SERDs significantly inhibited tumour growth (*p* = 0.007 and 0.047 for AZD9496 and fulvestrant, respectively). Depriving the tumours of E2 was sufficient to significantly block tumour growth, and the addition of fulvestrant or AZD9496 did not further enhance tumour growth inhibition (*p* = 0.232 and 0.305, respectively) (Fig. 2a). IHC staining showed limited changes in ER expression when fulvestrant or AZD9496 were administrated in the presence of E2 (Fig. 2b). In contrast, in the ED condition, ER expression was significantly reduced by AZD9496 and fulvestrant (Fig. 2b). ED also led to a substantial decrease in PR levels, and no agonistic activity by AZD9496 or fulvestrant treatment was observed (Fig. 2c). Gene expression profiling of 45 ER-regulated genes (Supplementary Table 2) in E2-stimulated tumours showed that both AZD9496 and fulvestrant significantly modulated the expression of 26 of the 45 genes tested (Fig. 2d); among these genes were *AREG, PGR, CXCL12, GREB1, MYC, LITAF*, and *BAMBI*. Of note, the two SERDs also relieved the E2 inhibitory effect on genes potentially involved in mechanisms of resistance, such as *ERBB2*^13^ and *TFF3*^24^. Only a limited number of genes (7 genes) were differentially modulated by AZD9496 compared to fulvestrant (Supplementary Fig. 1). These in vivo results suggest that both SERDs similarly target E2-mediated tumour growth and signalling.

### AZD9496 inhibits ER-dependent growth of EDR and TamR cells in vitro but is cross-resistant to fulvestrant

**Fig. 3:**
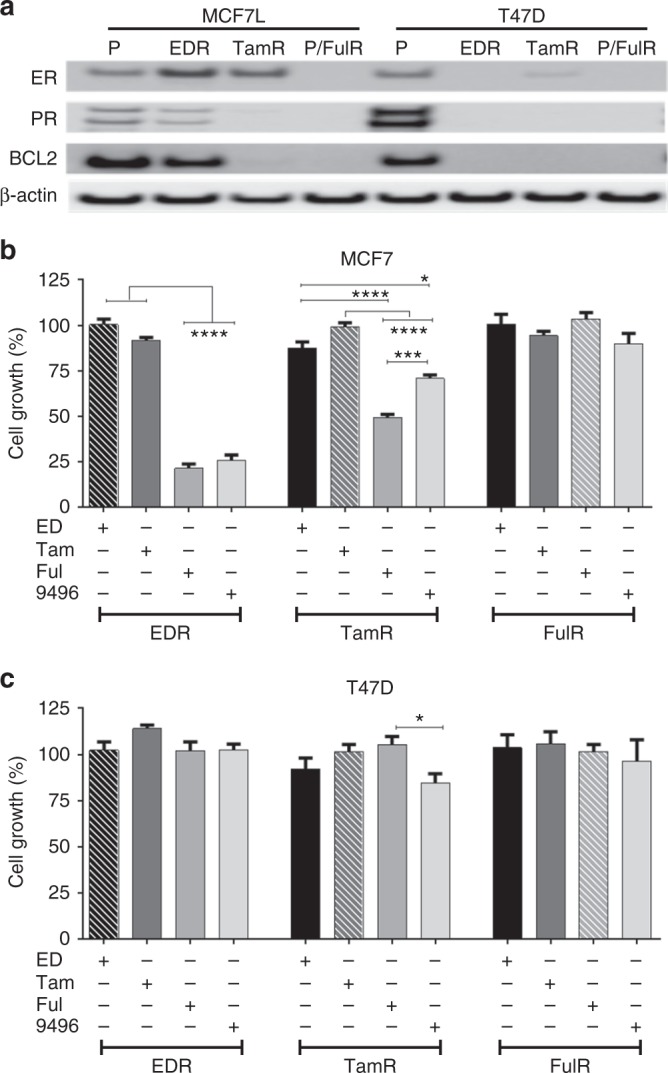
AZD9496 effect in endocrine-resistant ER +  cells. **a** Immunoblot for ER and downstream ER targets PR and BCL2 in MCF7 and T47D parental (P) and endocrine-resistant models (EDR, TamR, and FulR). **b**, **c** Effect of different endocrine therapies, all in ED-medium, ED, tamoxifen 10^–7^ M (Tam), fulvestrant 10^–7^ M (Ful), and AZD9496 10^–7^ M (9496), on the growth of resistant cell models: MCF7 EDR, TamR, and FulR (**b**); T47D EDR, TamR, and FulR (**c**). Cell viability is expressed as relative percentage compared to its own control (hatched bars; EDR to ED, TamR to Tam, and FulR to Ful) at day 6 of treatment. SEM are shown (*n* = 3); *p < 0.05; ***p < 0.001; ****p < 0.0001

We next evaluated the ER dependence of MCF7 and T47D cells and their endocrine-resistant derivatives including EDR (to mimic aromatase inhibitor resistance), TamR, and FulR. MCF7 EDR and TamR models retained ER expression; however, the classic ER target genes, such as PR and BCL2, were downregulated in EDR and lost in TamR compared to parental cells (Fig. 3a). In the T47D models, the TamR cells displayed low levels of ER expression and lacked PR and BCL2 expression. The T47D EDR and FulR cells lost expression of ER and classic ER target proteins (Fig. 3a). Silencing of ER expression with two different small interfering RNA (siRNAs) targeting ER^12^ demonstrated a profound growth inhibition (≥60%) in the MCF7 EDR and TamR models (Supplementary Fig. 2A-B), whereas in the MCF7 FulR model and in the T47D-resistant models in which ER expression was very low or undetectable, only minimal (MCF7 FulR) or no (all T47D endocrine-resistant derivatives) growth inhibition was observed upon targeting ER (Supplementary Fig. 2C-F).

We then tested AZD9496 in comparison with ED, tamoxifen, and fulvestrant in all the endocrine-resistant cell models. In the MCF7 EDR model, AZD9496 and fulvestrant but not tamoxifen markedly inhibited cell growth (Fig. 3b, and Supplementary Fig. 3A). In the MCF7 TamR model, fulvestrant and AZD9496 inhibited cell growth and ER expression, albeit the inhibitory effect of AZD9496 was less robust than that of fulvestrant (Fig. 3b and Supplementary Fig. 3B). The MCF7 FulR (Fig. 3b) and T47D EDR and FulR (Fig. 3c) derivatives displayed loss of ER and were resistant to all endocrine treatments, including AZD9496 and fulvestrant. The T47D TamR cells had low levels of ER. While fulvestrant did not have an inhibitory effect on these cells, AZD9496 modestly inhibited T47D TamR cell growth (Fig. 3c). In this model, further reductions of the already low levels of ER protein were seen with fulvestrant and AZD9496 (Supplementary Fig. 3C). These in vitro data suggest that AZD9496 can overcome endocrine resistance in models that remain ER dependent.

### AZD9496 overcomes ER-dependent growth in in vivo models of endocrine resistance

**Fig. 4:**
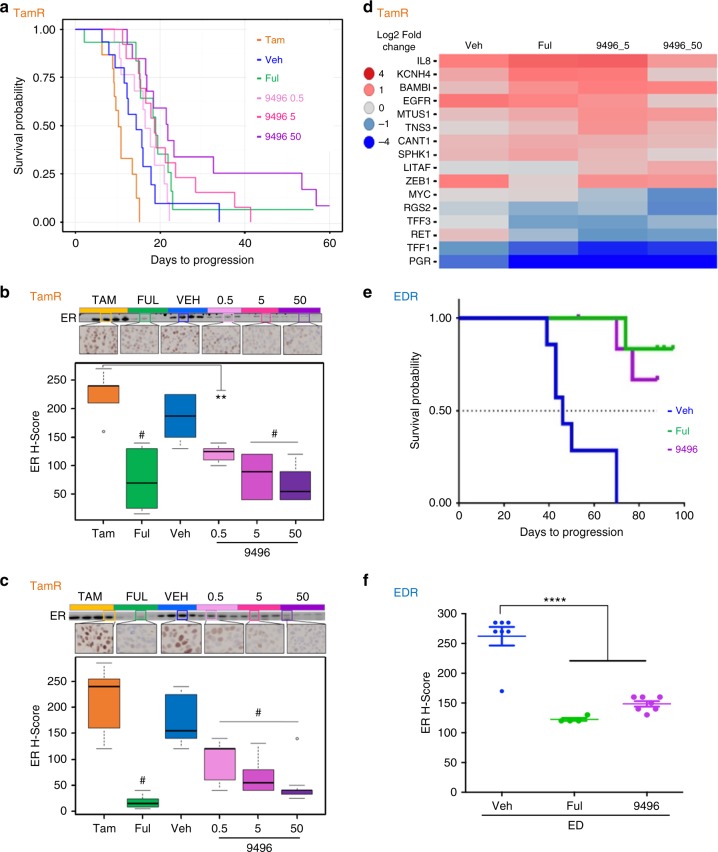
AZD9496 is comparable to fulvestrant in delaying tumour growth and reducing ER level in endocrine-resistant tumours in vivo. Effect of AZD9496 and fulvestrant on in vivo transplantable model of MCF7 TamR (**a**–**d**, orange boxes) and MCF7 EDR (**e**, **f**, blue boxes). **a** Kaplan–Meier curves showing the tumour tripling time of MCF7 TamR tumours. Tumour volume is assessed in the presence of tamoxifen control (Tam), stop tamoxifen treatment and switch to drug vehicle (Veh), fulvestrant (Ful), or 3 different doses of AZD9496 (9496), 0.5, 5, and 50 mg/kg (for each group *n* = 12). Data are reported as change of the average of tumours in the same group. **b** ER protein levels by western blot and IHC (highlighted square in the western blot for the same sample) of representative short-term (10 days) treated tumours with quantification of ER protein level by IHC using H-score. **c** ER protein levels in representative long-term treated TamR tumours and quantification of ER protein level of the long-term treated tumours by IHC using H-score. **d** Heat map of significant differentially expressed genes in at least one of the treated groups compared to the tamoxifen control group. **e** Kaplan–Meier curves of transplantable MCF7 EDR tumours in the presence of vehicle (Veh; *n* = 7), fulvestrant (Ful; *n* = 6), or AZD9496 (9496; *n* = 6) for tumour tripling time from baseline followed up for about 90 days (±1–2 weeks). **f** Quantification of ER protein level by IHC using H-score. SEMs are shown; **p < 0.01; ****p = < 0.0001, ^#^*p* < 0.01 compared to Tam and Veh, respectively. Cuzick test on (**b** and **c**) for Veh, 0.5, 5, and 50 mg/kg AZD9496 (not shown) *p* < 0.0001

We next studied the dose-dependent effect of AZD9496 using three doses (0.5, 5, and 50 mg/kg) and a standard fulvestrant dose (5 mg) in a transplantable MCF7 TamR in vivo developed model^20^. Kaplan–Meier assessment showed that AZD9496 and fulvestrant significantly delayed TamR tumour growth (Fig. 4a). Median time to tumour progression was 10 days for tamoxifen, 13 days for vehicle, 16.5 days for 0.5 mg/kg, 19 days for 5 mg/kg, and 22 days for 50 mg/kg AZD9496, and 19 days for fulvestrant, with a *p* value of ≤0.03 for all SERD treatments compared to tamoxifen. No difference was observed in median time to tumour progression between 5 mg/kg and 50 mg/kg AZD9496 (Fig. 4a). Fulvestrant and AZD9496 significantly reduced ER levels as tested by immunohistochemistry and western blot (Fig. 4b, c). The effect of short- (Fig. 4b, 10 days) or long-term AZD9496 treatment (Fig. 4c) on ER expression was dose dependent, suggesting an on-target effect (Cuzick nonparametric test, *p* ≤ 0.0001). Expression analysis of ER-modulated genes (Supplementary Table 2) in short-term treatment-sensitive tumours showed that fulvestrant and AZD9496, at 5 and/or 50 mg/kg, significantly modulated ER-dependent gene expression in comparison to tamoxifen (Fig. 4d). The overall pattern of gene expression was comparable between the two SERDs, with only 5 genes significantly modulated by both SERDs, one upregulated (*KCNN4*) and four downregulated (*RET, TFF1*, *TFF3*, and *PGR*) (Supplementary Fig. 4). These studies indicate that both SERDs alter gene expression of ER+ endocrine-resistant tumours, mostly blocking classic ER-induced gene transcription.

Since different TamR transplantable xenograft lines display different degrees of sensitivity to fulvestrant, we next conducted a study with another MCF7 TamR model that is more sensitive to fulvestrant and AZD9496. In this model, the median time to tumour progression was 26 and 69 days for tamoxifen and vehicle, respectively, 92 days for fulvestrant (*p* = 0.0021), and 75 days for AZD9496 (*p* = 0.0119) (Supplementary Fig. 5).

We next evaluated the long-term effects of AZD9496 using an in vivo developed MCF7 EDR transplantable model. Fulvestrant and AZD9496 significantly inhibited the growth of these xenografts. Tumour regression was observed in two fulvestrant-treated mice. Only one fulvestrant-treated and two AZD9496-treated tumours progressed during the 90-day treatment period (Fig. 4e). Both SERDs reduced ER protein level as demonstrated by quantification of IHC staining using H-score (Fig. 4f).

Overall, our studies suggest that the oral SERD AZD9496 is comparable to fulvestrant in the endocrine-resistant setting in inhibiting tumour growth, and in reducing ER levels and ER-regulated transcription.

## Discussion

Fulvestrant is the only Food and Drug Administration (FDA)-approved SERD. However, its low bioavailability and intramuscular route of administration are clinical limitations, raising the need for a novel oral SERD with a more favourable bioavailability profile. Several oral SERDs are currently in the early phase of clinical development. Unlike other oral SERDs, such as GDC-0810 (ARN-810)^25^ and RAD1901^26^, or mixed SERM/SERD Hybrid drugs^27^, AZD9496 was developed from a direct ER binding screen to identify new motifs with drug-like properties, which could degrade ER. As such, AZD9496 has a structure very similar to that of E2 when bound to ER^7^. Preclinical studies have shown the activity of AZD9496 mainly in ER+ endocrine-sensitive cell lines and a limited number of resistant models. Here, we expanded upon previous studies and investigated the activity of AZD9496 in comparison to fulvestrant in a number of ER+ endocrine-resistant models including models of oestrogen deprivation resistance, tamoxifen resistance, and fulvestrant resistance. In addition, in endocrine-sensitive models we comprehensively compared the activity of AZD9496 to oestrogen deprivation, tamoxifen, and fulvestrant.

Here we show that the oral SERD AZD9496 displayed similar efficacy to fulvestrant in inhibiting ER+ endocrine-sensitive breast cancer cell growth, and that both SERDs were equivalent or superior to tamoxifen and ED. Of note, in the short-term in vivo study with MCF7 xenografts, in the presence of E2, both SERDs significantly modulated ER-dependent gene expression, including E2-induced and -repressed genes involved in cell growth and potentially in escape pathways of endocrine resistance^13,24^.

In the endocrine-resistant models, our data show that both SERDs inhibit cell growth only in models that have retained a substantial level of dependency on ER, as shown by parallel experiments using genomic ER degradation by siRNA. These studies suggest that the inhibitory effect of the SERDs is ER-mediated and not due to off-target effects. Moreover, using our transplantable in vivo developed MCF7 EDR model, we showed that both fulvestrant and AZD9496 reduced tumour growth and ER protein level, with no significant difference between the two SERDs. These results are in agreement with our in vitro data and a previously reported in vivo study using an in vitro developed EDR model^8^.

The transplantable MCF7 TamR lines have a degree of heterogeneity in response to fulvestrant that can be attributed to some drift in the tumourigenic population selected with each transplantation, explaining the increased sensitivity to both SERDs in our second experiment. Importantly, however, the effect of AZD9496 on tumour growth was comparable to that of fulvestrant in both experiments, showing for the first time that indeed the oral SERD AZD9496 is as effective as fulvestrant in the TamR setting in vivo. In order to better understand the pharmacology of AZD9496 in the TamR model, a biomarker analysis after short-term (10-day) treatment was conducted. The RNA profiling of 45 ER-regulated genes (Supplementary Table 2) revealed that short-term AZD9496 and fulvestrant treatments both effectively inhibited the residual expression of classic E2-induced genes (such as *PGR* and *TFF1*), further suggesting that both SERDs inhibited tumour growth, at least partly, by effective degradation and/or blockade of ER transcriptional activity.

Although we detected a growth inhibitory effect in the TamR models with AZD9496 and fulvestrant treatment, we did not observe tumour regression after short- or long-term fulvestrant or AZD9496 despite continued ER repression. These findings are likely due to incomplete degradation of ER and/or the activation of other pathways of resistance. This suggests that future studies should include therapeutic strategies with combinations with other targeted treatments. One possibility would be the combination of a CDK4/6 inhibitor and AZD9496. The PALOMA3^28^ trial showed that addition of the CDK4/6 inhibitor palbociclib to fulvestrant resulted in doubling of the median progression-free survival compared to fulvestrant alone, and led to the FDA approval of palbociclib in combination with fulvestrant for ER+ metastatic breast cancer. In agreement with this notion, in a recent preclinical model, AZD9496 has been shown to induce tumour regression when combined with palbociclib or inhibitors of the PI3K pathway^8^.

In this study we also investigated the activity of AZD9496 in fulvestrant-resistant models. The fulvestrant-resistant models were resistant to AZD9496, suggesting cross-resistance between AZD9496 and fulvestrant. The cross-resistance we detected in preclinical studies will need to be investigated in the clinical setting, as the oral bioavailability of AZD9496 may provide activity even in the setting of resistance to fulvestrant treatment. Similarly, our in vivo studies show overall comparable activity between AZD9496 and fulvestrant in both endocrine-sensitive and -resistant models. However, the oral bioavailability of AZD9496 may result in improved clinical benefit. The ongoing pre-surgical window-of-opportunity studies that will compare the pharmacodynamic endpoints of AZD9496 versus fulvestrant will hopefully shed light on this question (NCT03236974).

## Electronic supplementary material

Supplementary Methods and figures legend

Supplementary Fig 1

Supplementary Fig 2

Supplementary Fig 3

Supplemenatry Fig 4

Supplementary Fig 5

Supplementary Table 1

Supplementary Table 2
