# Supplementary material for: The oral selective oestrogen receptor degrader (SERD) AZD9496 is comparable to fulvestrant in antagonising ER and circumventing endocrine resistance
Source: Br J Cancer. 2018 Dec 17;120(3):331–9. doi: 10.1038/s41416-018-0354-9 (PMC6353941; doi:10.1038/s41416-018-0354-9)
Supplement: Supplementary file 1 — Supplementary Methods and figures legend [file 41416_2018_354_MOESM1_ESM.docx]

**Supplementary Methods**

**siRNA Transfection**

Small-interfering RNA (siRNA) oligos targeting ERα, along with non-targeting siRNA, were purchased (Ambion, and Dharmacon). Cells were transfected with siRNA by reverse transfection per the manufacturers' directions as previously described [1]. Briefly, 2.5-5x10^3^ cells/well were seeded into 96-well plates containing a pre-incubated mixture of single siRNA oligos at 5 nM final concentration and Lipofectamine RNAiMax (Invitrogen) diluted in Opti-MEM (Invitrogen) as per the manufacturers' directions. The appropriate cell-specific medium supplemented with the relevant, respective drugs was added 24 hours after transfection and the effect of siRNA was determined after an additional 6 days (5 days after changing media). For parallel protein expression analysis, 5x10^5^ cells/well were plated into six-well plates, subjected to the transfection protocol as above, and harvested 48hrs after transfection.

**Xenograft Studies**

**TamR study.** MCF7 TamR xenograft tumors were generated and maintained as described in the TamR study in the Methods. Mice were transplanted on both sides with tumors derived from two independent donors. When at least one of the two tumors reached 150-200 mm^3^, mice were randomized to continue tamoxifen (Tam) or stop tamoxifen and switch to vehicle (PEG/Captisol), fulvestrant (5mg once a week s.c., with an extra dose in the first week), or AZD9496 (10 mg/Kg by oral gavage daily). Tumor volumes were measured weekly as previously described. When tumors reached 1000 mm^3^ in volume, they were harvested 24 hours after the last fulvestrant treatment or 4 hours after the final vehicle or AZD9496 treatment. The average of both tumors for the same mouse was used for the statistical analysis.

**Supplementary Figures**

**
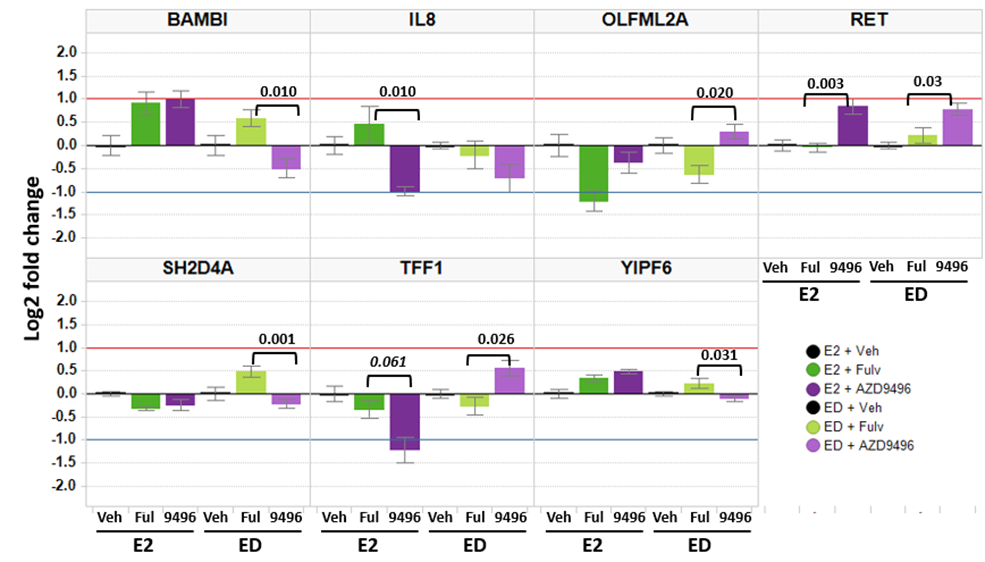
**

**Supplementary Figure 1: Genes differentially modulated between fulvestrant and AZD9496 in MCF7 parental *in vivo* model.** Bar chart of genes differentially modulated between fulvestrant and AZD9496, in the presence of E2 or ED. Statistical p-values of the differences are reported. Data are represented as log2 fold change — log2 fold change of 1 or -1 corresponds to 2 or -2 fold change, marked by red or blue lines respectively.


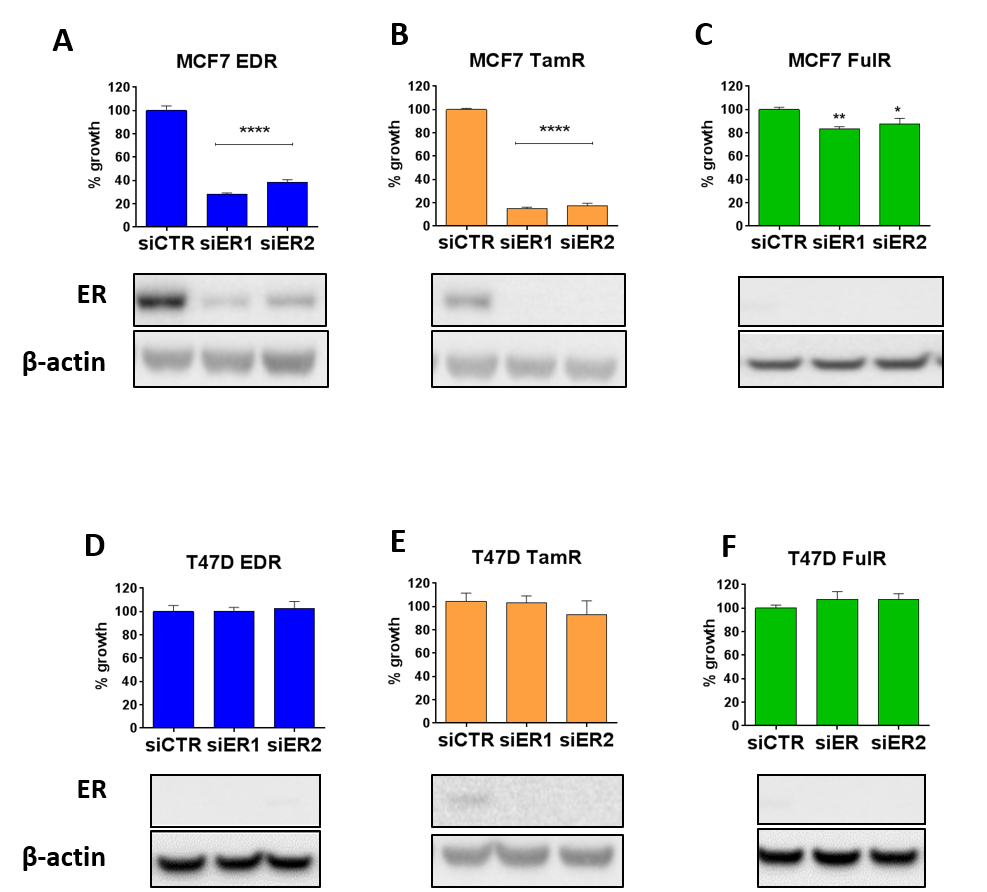


**Supplementary Figure 2:** **ER inhibition in endocrine-resistant ER+ cell lines**. Cell growth and ER protein level effect of two ER siRNA sequences on MCF7 EDR (A), TamR (B), and FulR (C) and on T47D EDR (D), TamR (E), and FulR (F)**.** β-actin was used as loading control. *, p<0.05; **, p<0.01; ****, p<0.0001.


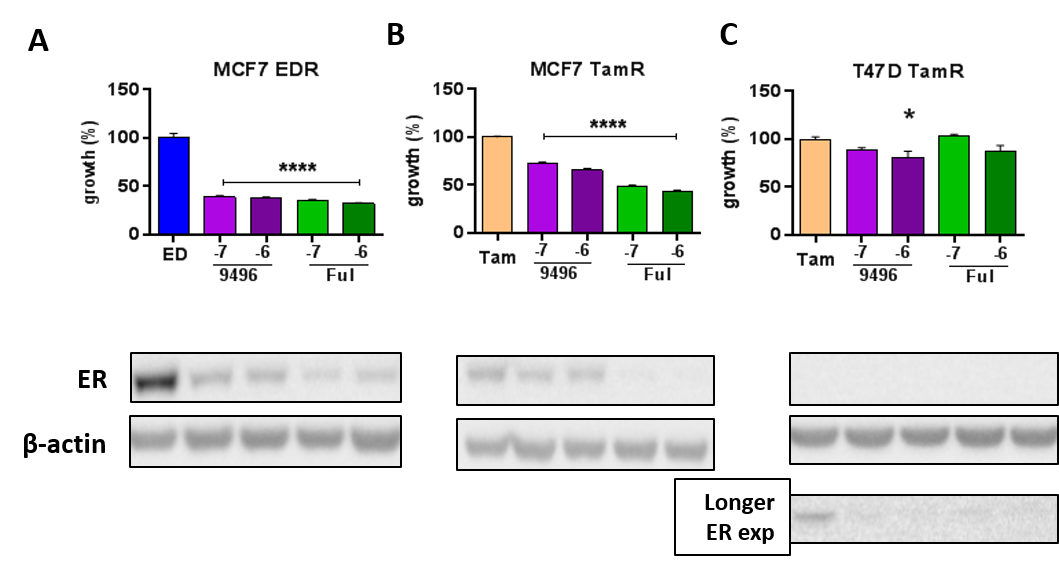


**Supplementary Figure 3: Effect of different concentrations of AZD9496 and fulvestrant in ER+ resistant cell lines.** Cell growth and ER protein levels of two different concentrations (10^-6^ and 10^-7^ M) of AZD9496 or fulvestrant on MCF7 EDR (A) and TamR (B), and on T47D TamR (C), in comparison with their own control (ED for EDR and Tam for TamR). β-actin is used as loading control. *, p<0.05; ****, p<0.0001.

**
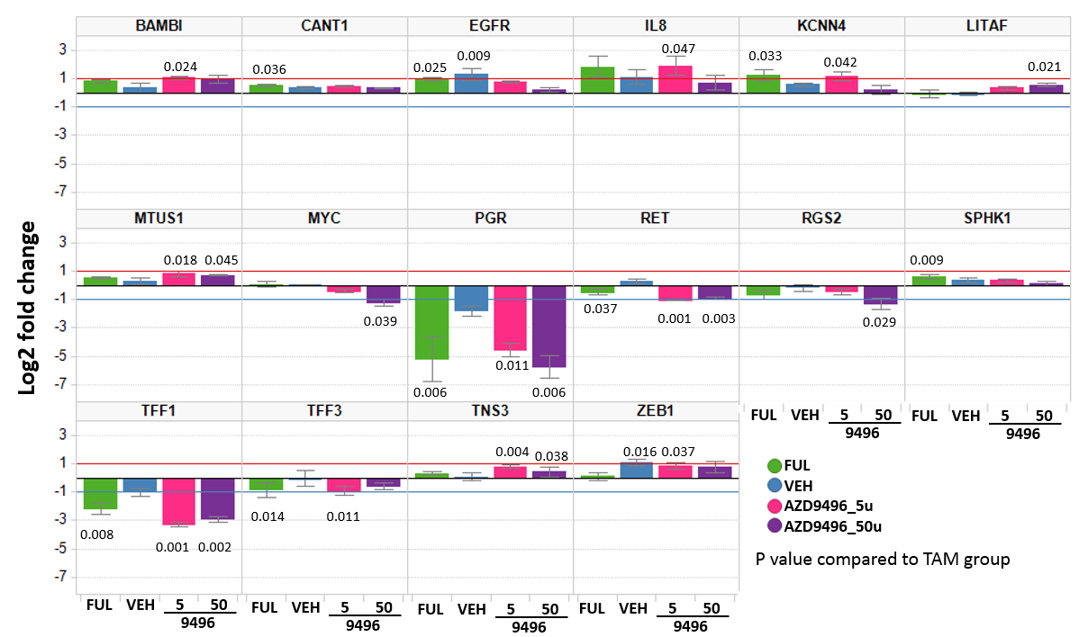
**

**Supplementary Figure 4: Genes modulated in the MCF7 TamR *in vivo* model.** Bar chart of genes differentially modulated, by fulvestrant and/or AZD9496 5 and 50mg/kg. Statistical p-values of the differences are reported. Data are represented as log2 fold change — log2 fold change of 1 or -1 corresponds to 2 or -2 fold change, marked by red or blue lines respectively.

**
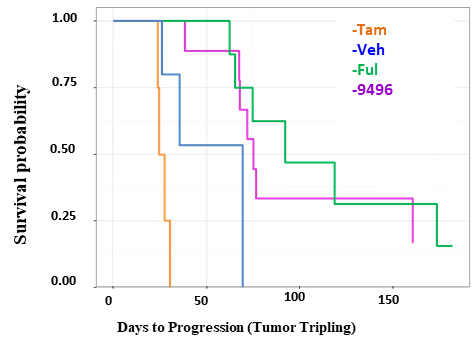
**

**Supplementary Figure 5: AZD9496 is comparable to fulvestrant in delaying tumor growth in TamR tumors.** Kaplan-Meier curve for tumor tripling time from baseline of MCF7 TamR xenograft tumors randomized to continue tamoxifen or to be switched to vehicle, fulvestrant, or AZD9496.


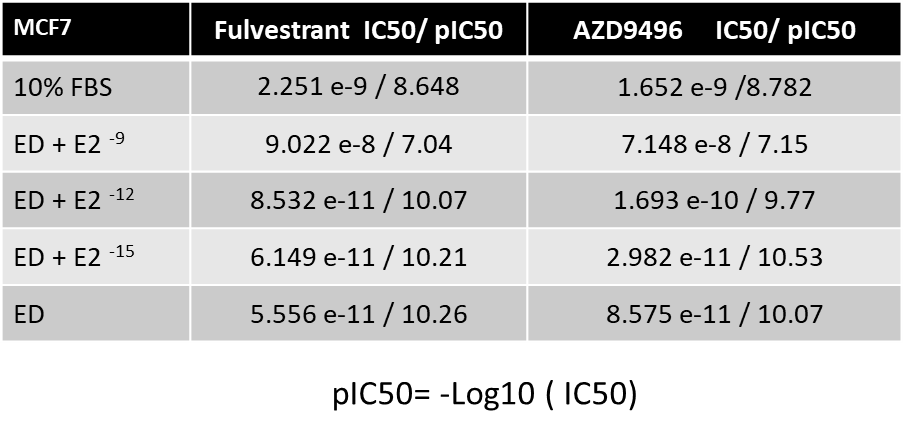


**Supplementary Table 1: IC50 of fulvestrant and AZD9496 in presence of increased concentrations of E2.** MCF7 parental cells were starved in ED media and treated with DMSO or increasing concentrations of fulvestrant and AZD9496 in presence of endogenous (10% FBS) or exogenous E2 (10^-9^/10^-15^ M) and ED media. IC50 and pIC50 [-log10 (IC50)] in the different conditions are reported.


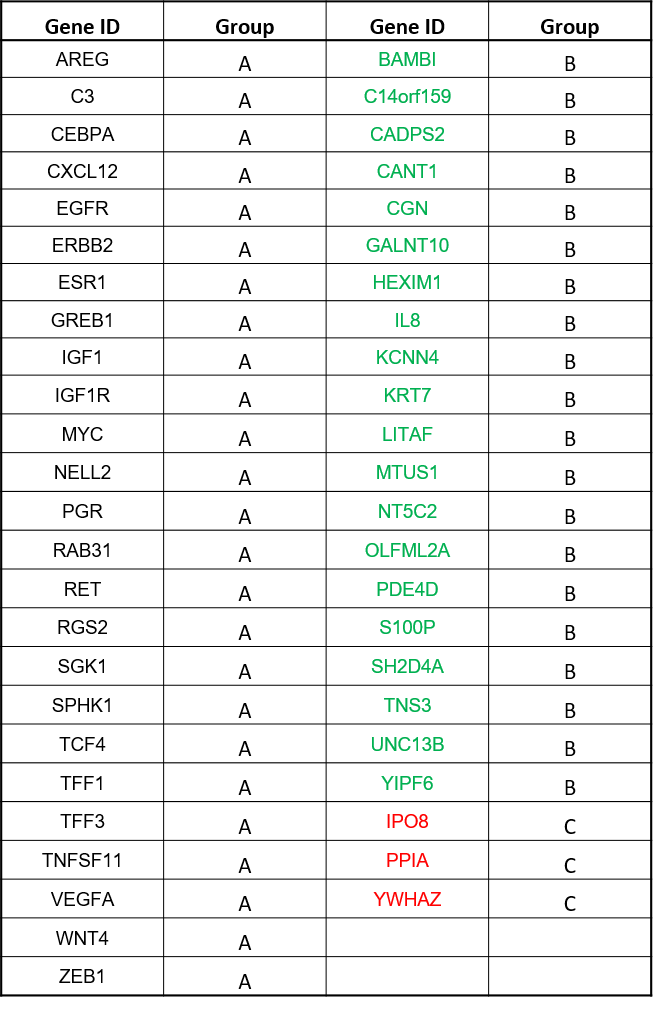


**Supplementary Table 2: FLUIDIGM ER-dependent genes.** List of the 48 genes tested. Group A: Selected ER-regulated genes taken from a computer-based analysis of all literature where gene expression data had been generated for a number of endocrine therapies (AstraZeneca, unpublished data). Group B: A subset of the 93 genes we have previously described [2] (genes modulated in MCF7/TamR xenografts that are associated with the growth factor-induced oestrogen receptor (ER) cistrome, and harbor an AP-1 motif within 20 Kb of their transcription start site; Figure 1C of [2]) that are also differentially expressed in and enriched in the ER cistrome of our MCF7/TamR cell line model in comparison to parental cells [3]. Group C: Housekeeping genes.

**Supplementary References:**

1. Wang YC, Morrison G, Gillihan R, Guo J, Ward RM, Fu X, Botero MF, Healy NA, Hilsenbeck SG, Phillips GL *et al*: **Different mechanisms for resistance to trastuzumab versus lapatinib in HER2-positive breast cancers--role of estrogen receptor and HER2 reactivation**. *Breast Cancer Res* 2011, **13**(6):R121.

2. Malorni L, Giuliano M, Migliaccio I, Wang T, Creighton CJ, Lupien M, Fu X, Hilsenbeck SG, Healy N, De Angelis C *et al*: **Blockade of AP-1 potentiates endocrine therapy and overcomes resistance**. *Mol Cancer Res* 2016, **14**(5):470-481.

3. Jeselsohn R, Cornwell M, Pun M, Buchwalter G, Nguyen M, Bango C, Huang Y, Kuang Y, Paweletz C, Fu X *et al*: **Embryonic transcription factor SOX9 drives breast cancer endocrine resistance**. *Proc Natl Acad Sci U S A* 2017, **114**(22):E4482-E4491.
