# Supplementary figures and images for: The oral selective oestrogen receptor degrader (SERD) AZD9496 is comparable to fulvestrant in antagonising ER and circumventing endocrine resistance
Source: Br J Cancer. 2018 Dec 17;120(3):331–9. doi: 10.1038/s41416-018-0354-9 (PMC6353941; doi:10.1038/s41416-018-0354-9)

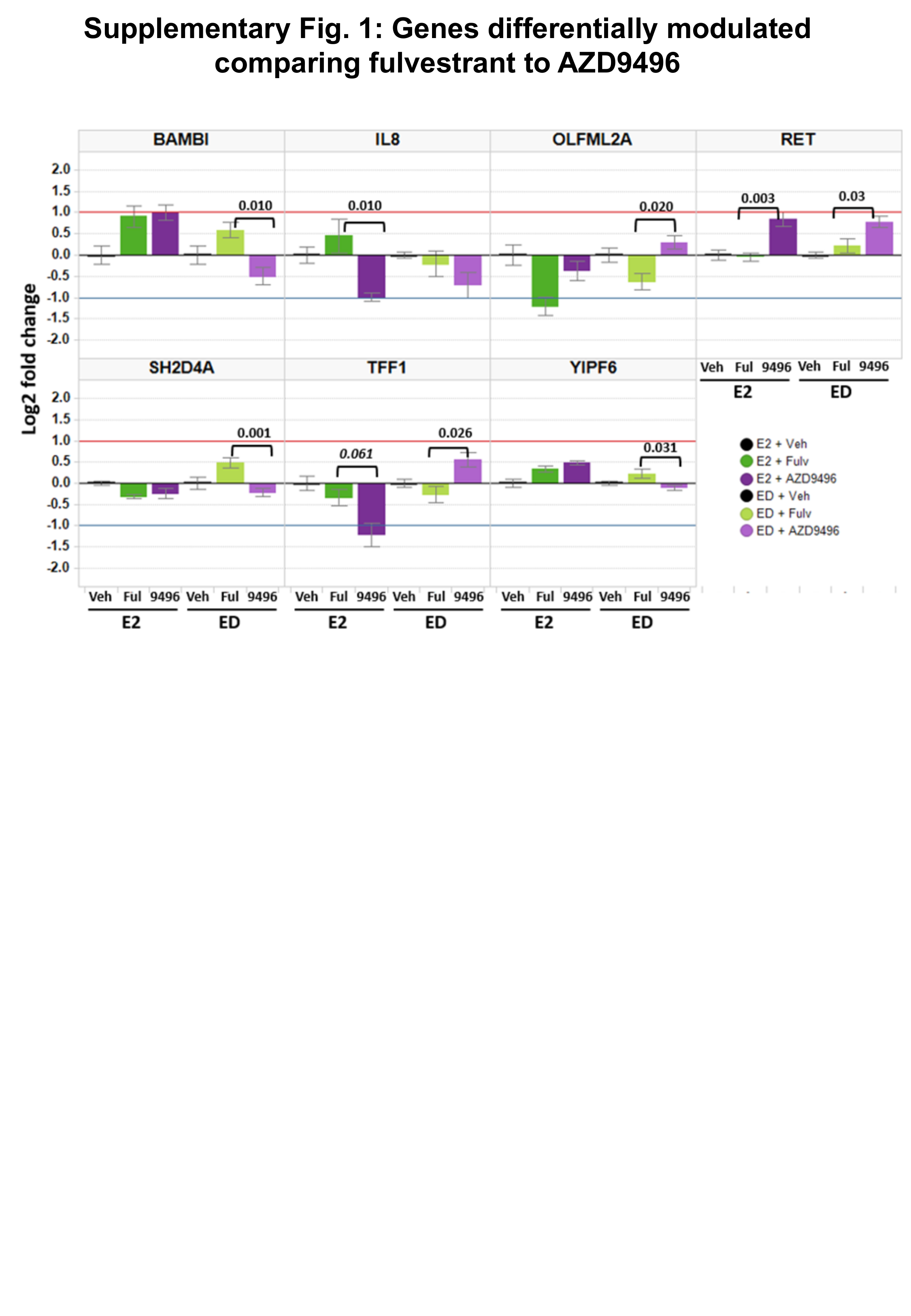

Supplement: Supplementary file 2 — Supplementary Fig 1 [file 41416_2018_354_MOESM2_ESM.tif]

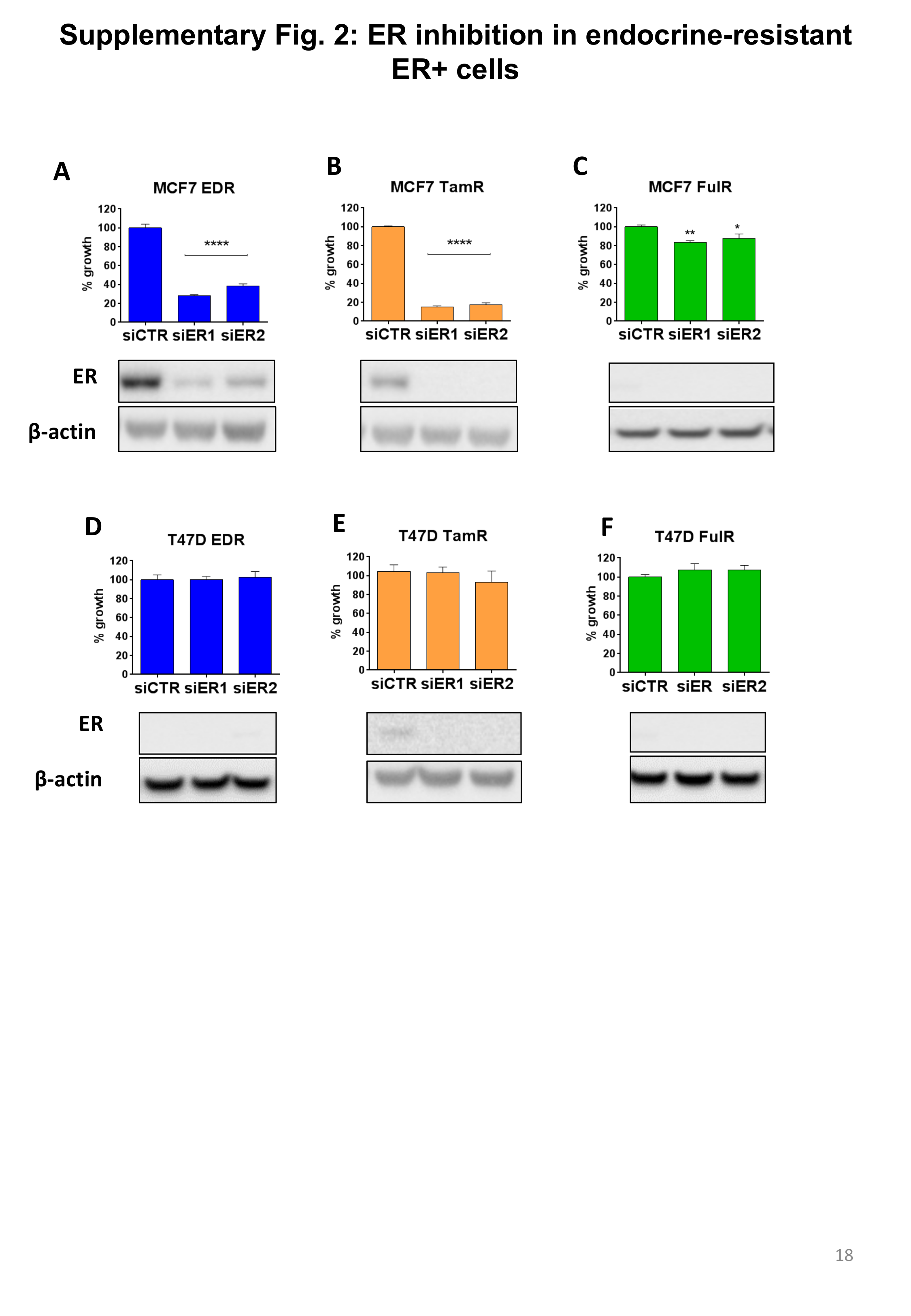

Supplement: Supplementary file 3 — Supplementary Fig 2 [file 41416_2018_354_MOESM3_ESM.tif]

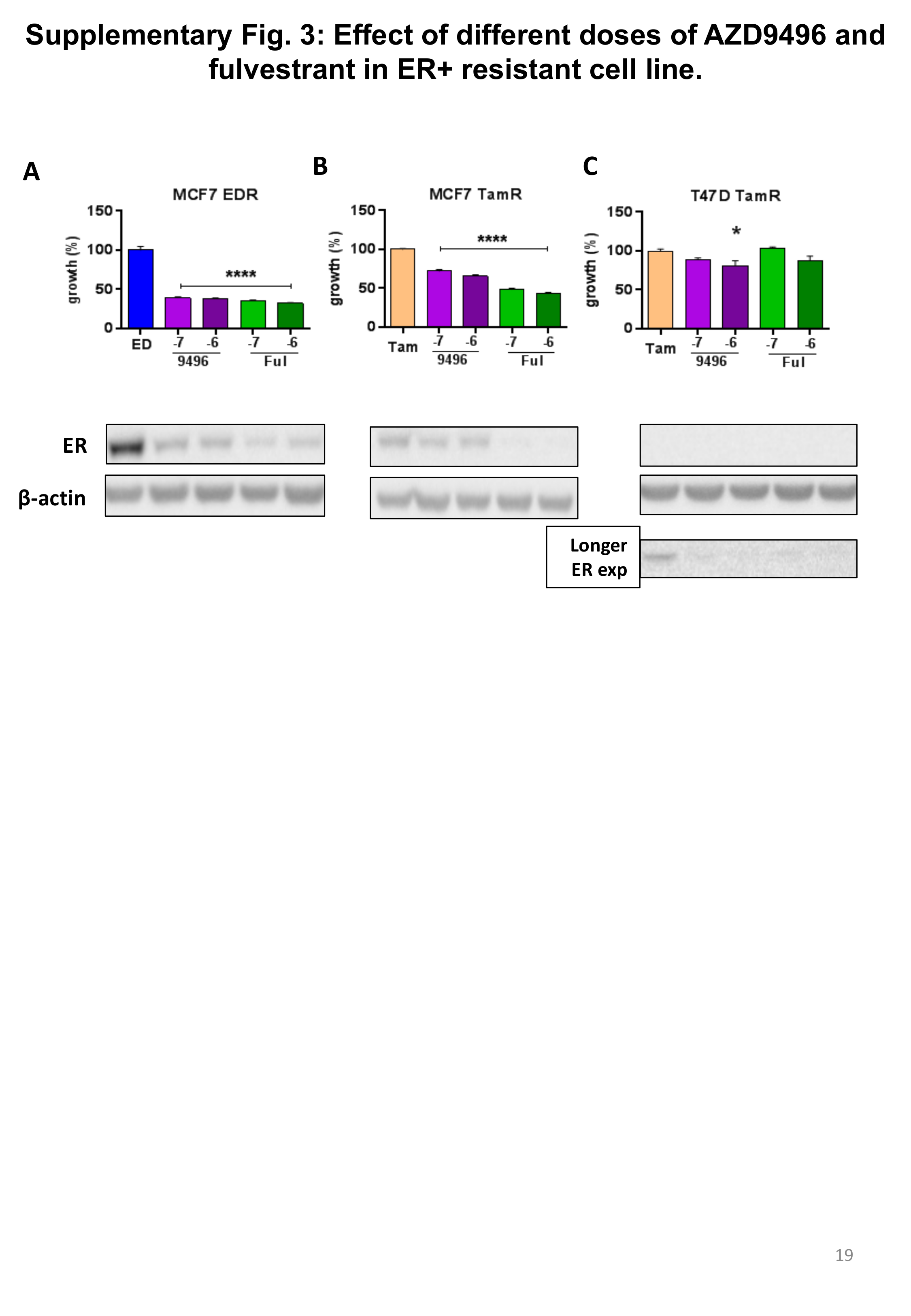

Supplement: Supplementary file 4 — Supplementary Fig 3 [file 41416_2018_354_MOESM4_ESM.tif]

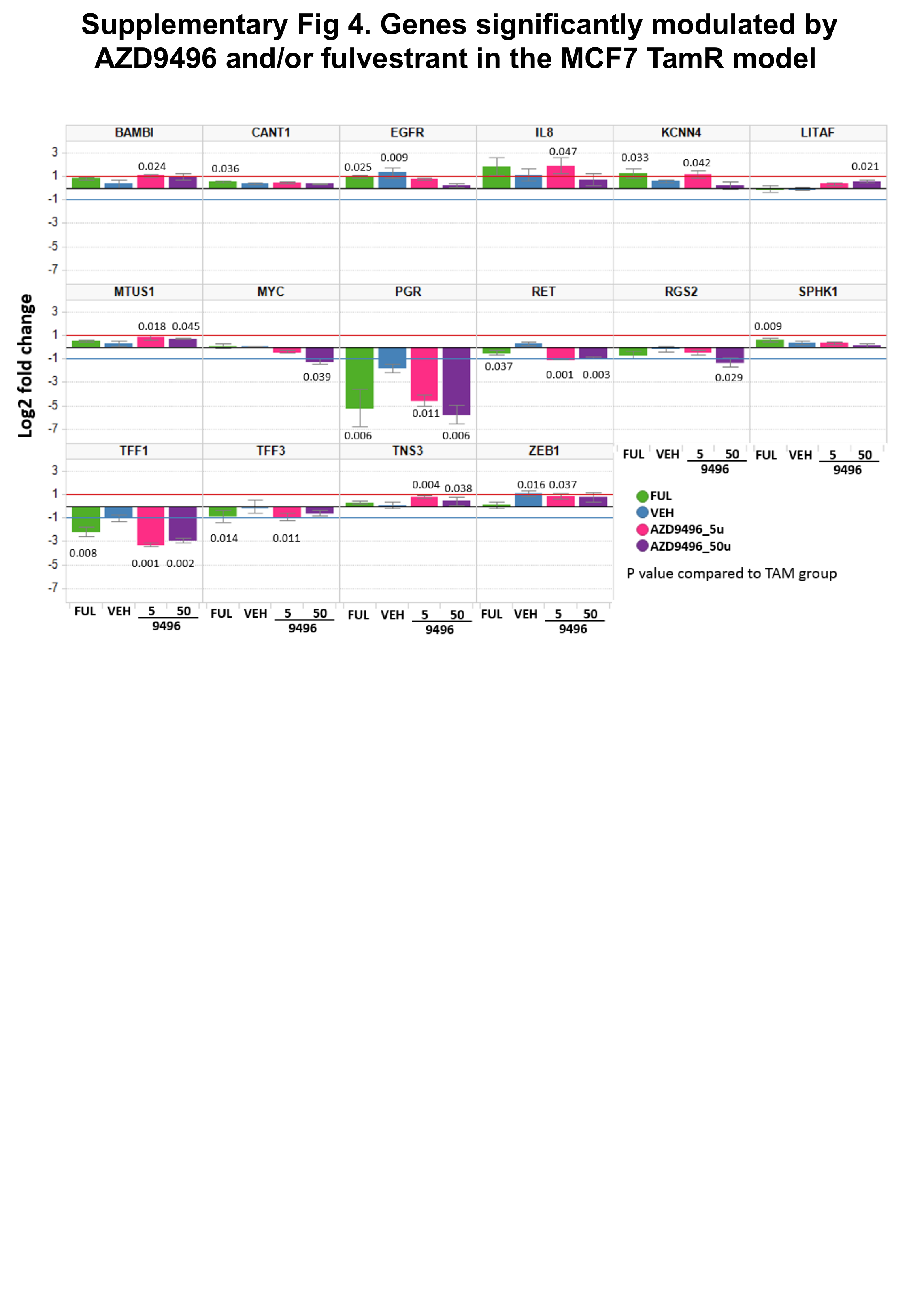

Supplement: Supplementary file 5 — Supplemenatry Fig 4 [file 41416_2018_354_MOESM5_ESM.tif]

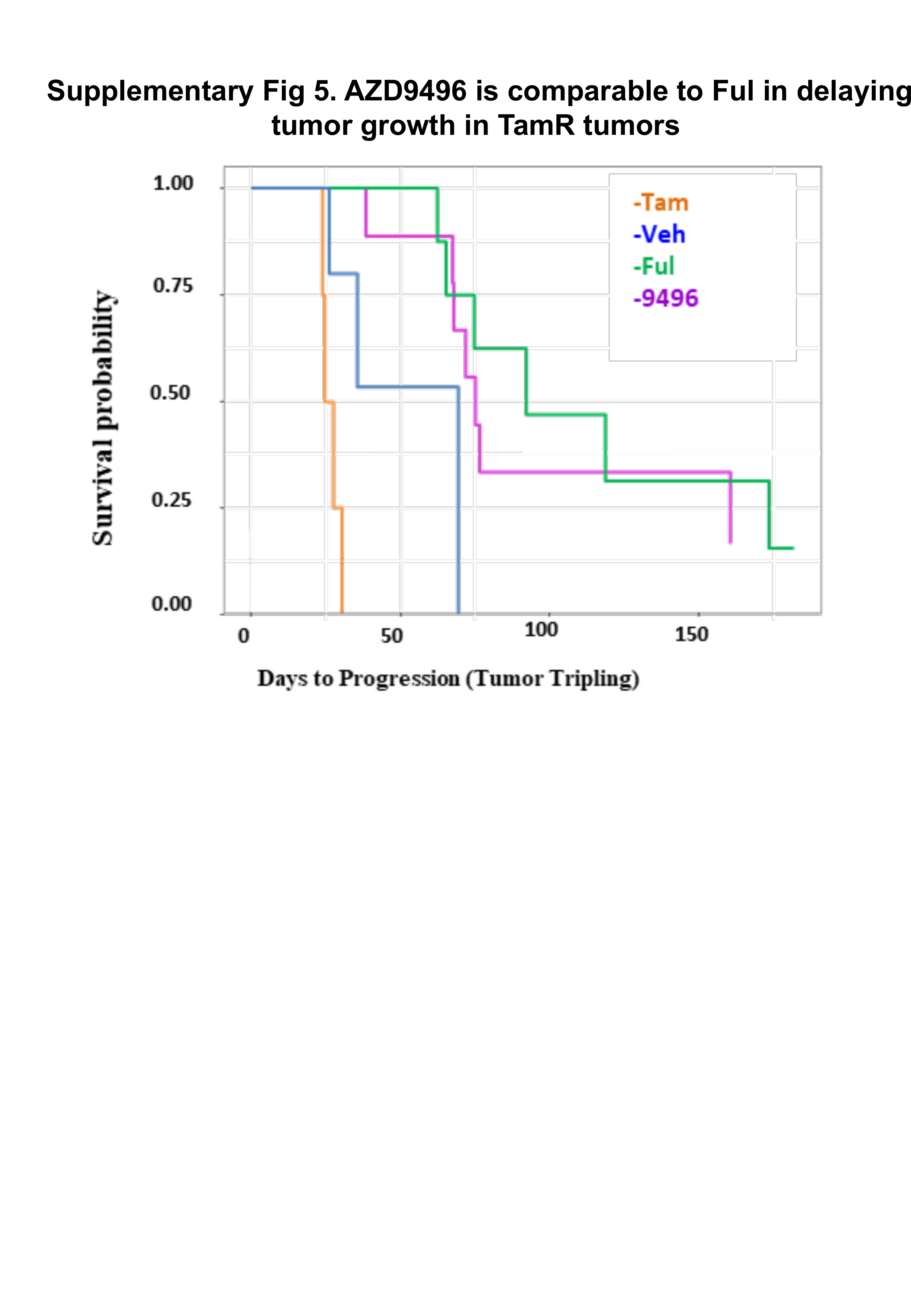

Supplement: Supplementary file 6 — Supplementary Fig 5 [file 41416_2018_354_MOESM6_ESM.tif]
