# Supplementary material for: The oral selective oestrogen receptor degrader (SERD) AZD9496 is comparable to fulvestrant in antagonising ER and circumventing endocrine resistance
Source: Br J Cancer. 2018 Dec 17;120(3):331–9. doi: 10.1038/s41416-018-0354-9 (PMC6353941; doi:10.1038/s41416-018-0354-9)
Supplement: Supplementary file 7 — Supplementary Table 1 [file 41416_2018_354_MOESM7_ESM.pptx]

## Slide 1
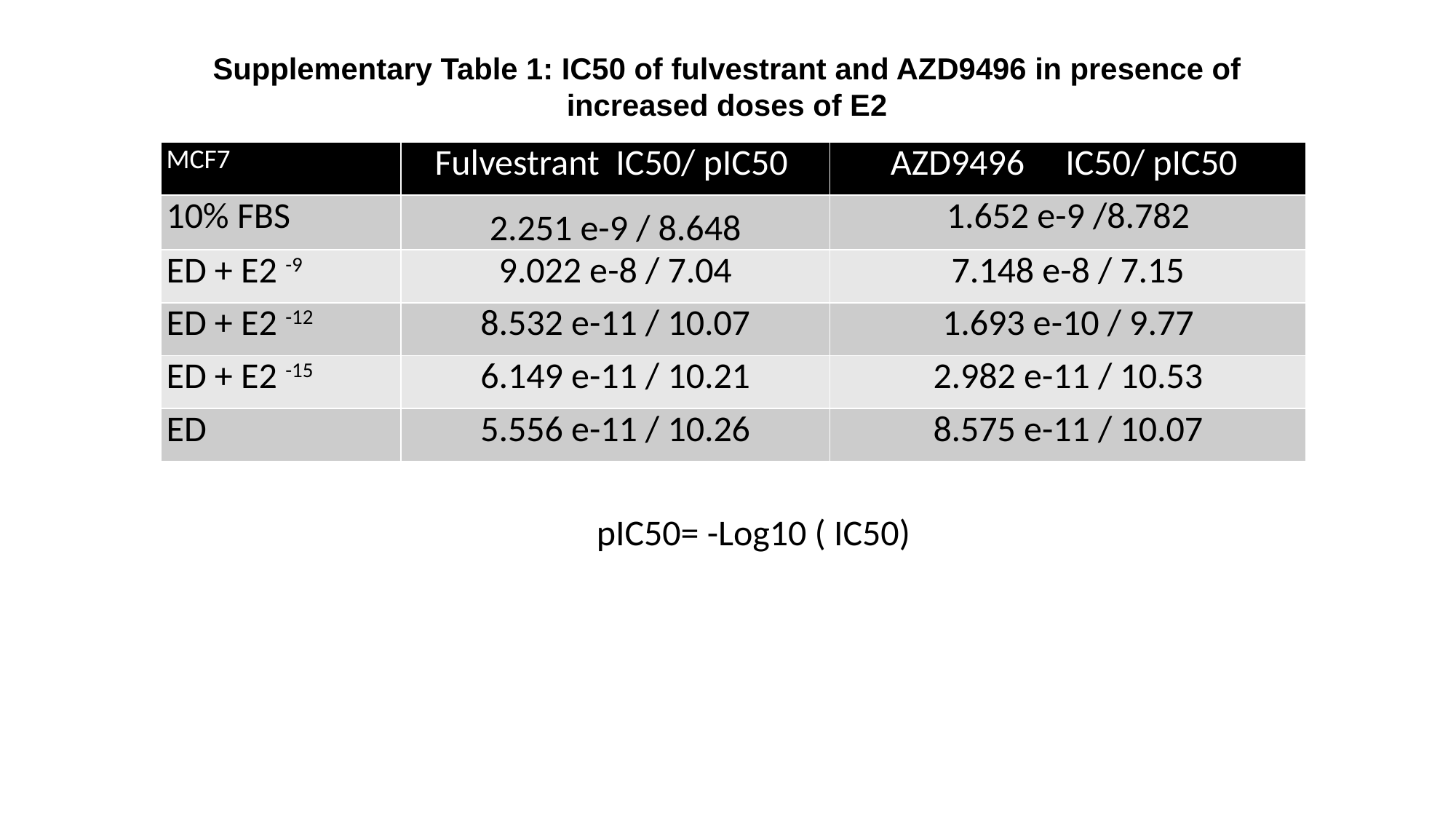

Supplementary Table 1: IC50 of fulvestrant and AZD9496 in presence of increased doses of E2
| MCF7 | Fulvestrant IC50/ pIC50 | AZD9496 IC50/ pIC50 |
| --- | --- | --- |
| 10% FBS | 2.251 e-9 / 8.648 | 1.652 e-9 /8.782 |
| ED + E2 -9 | 9.022 e-8 / 7.04 | 7.148 e-8 / 7.15 |
| ED + E2 -12 | 8.532 e-11 / 10.07 | 1.693 e-10 / 9.77 |
| ED + E2 -15 | 6.149 e-11 / 10.21 | 2.982 e-11 / 10.53 |
| ED | 5.556 e-11 / 10.26 | 8.575 e-11 / 10.07 |
pIC50= -Log10 ( IC50)
