# Supplementary material for: The oral selective oestrogen receptor degrader (SERD) AZD9496 is comparable to fulvestrant in antagonising ER and circumventing endocrine resistance
Source: Br J Cancer. 2018 Dec 17;120(3):331–9. doi: 10.1038/s41416-018-0354-9 (PMC6353941; doi:10.1038/s41416-018-0354-9)
Supplement: Supplementary file 8 — Supplementary Table 2 [file 41416_2018_354_MOESM8_ESM.pptx]

## Slide 1
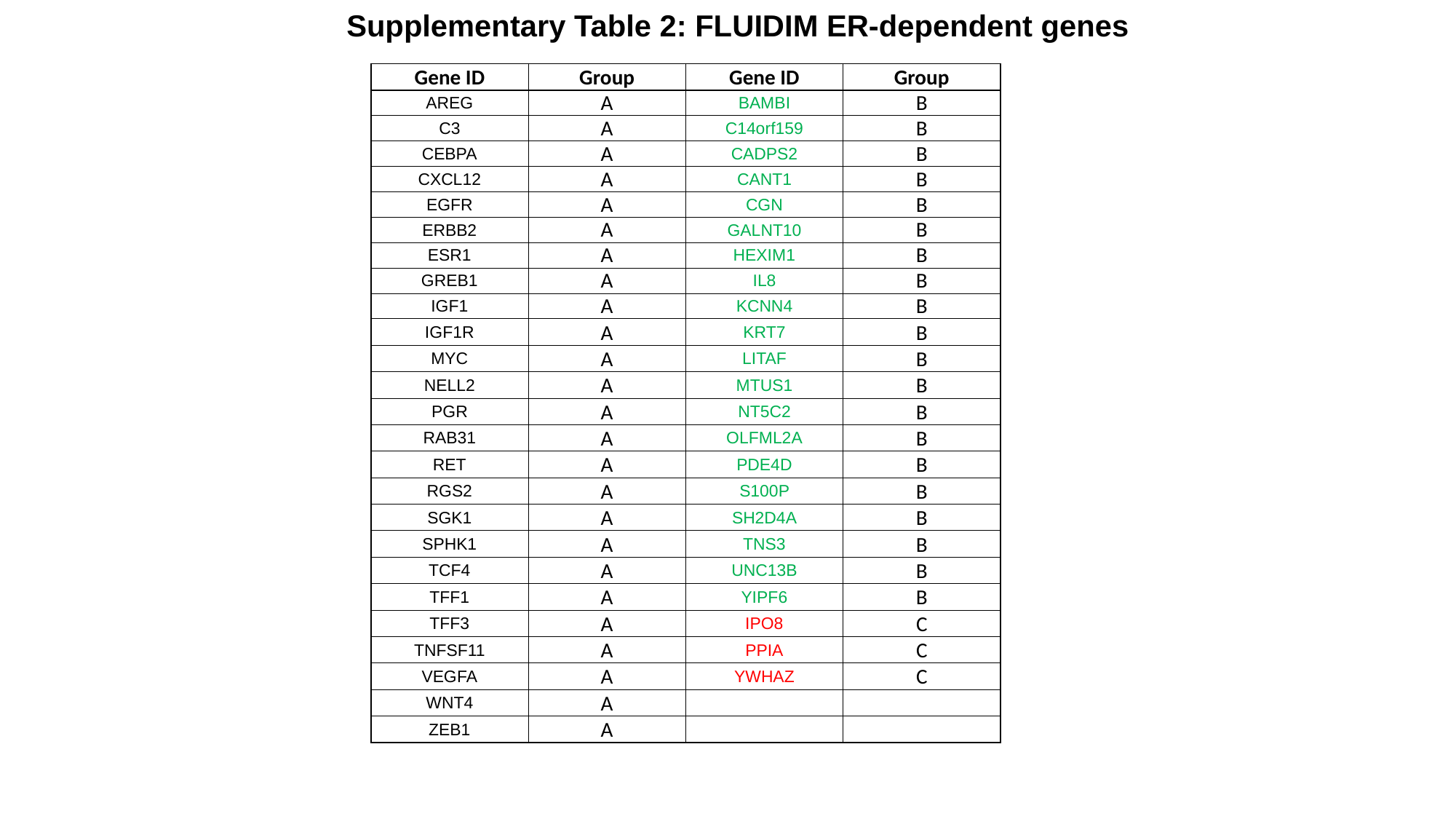

Supplementary Table 2: FLUIDIM ER-dependent genes
| Gene ID | Group | Gene ID | Group |
| --- | --- | --- | --- |
| AREG | A | BAMBI | B |
| C3 | A | C14orf159 | B |
| CEBPA | A | CADPS2 | B |
| CXCL12 | A | CANT1 | B |
| EGFR | A | CGN | B |
| ERBB2 | A | GALNT10 | B |
| ESR1 | A | HEXIM1 | B |
| GREB1 | A | IL8 | B |
| IGF1 | A | KCNN4 | B |
| IGF1R | A | KRT7 | B |
| MYC | A | LITAF | B |
| NELL2 | A | MTUS1 | B |
| PGR | A | NT5C2 | B |
| RAB31 | A | OLFML2A | B |
| RET | A | PDE4D | B |
| RGS2 | A | S100P | B |
| SGK1 | A | SH2D4A | B |
| SPHK1 | A | TNS3 | B |
| TCF4 | A | UNC13B | B |
| TFF1 | A | YIPF6 | B |
| TFF3 | A | IPO8 | C |
| TNFSF11 | A | PPIA | C |
| VEGFA | A | YWHAZ | C |
| WNT4 | A | | |
| ZEB1 | A | | |
